# Innovations in Economics of Aging: Household Financial Factors and Well-Being in Later Life

**DOI:** 10.1093/geroni/igaf122.1409

**Published:** 2025-12-31

**Authors:** Jessica Kelley, Dawn Carr

## Abstract

Income and wealth are broadly understood to shape what it means to age well. Other household financial factors, however, receive less attention and may have more daily impact on well-being. For instance, the daily strain of having sufficient food or managing debt in older adulthood can diminish cognitive, emotional, or physical health. This symposium brings together scholars who utilize innovative measures about financial constraints or challenges and their relation to later-life outcomes. Topics include: midlife food insecurity and SNAP receipt in midlife, neighborhood proximity to “bricks and mortar” banks, debt-to-asset ratios, and later life mortgage borrowing. Dawn Carr will serve as discussant as we explore ways to expand inquiry on the economic-health linkage over the life course.

